# Low muscle, high leak? The aMFR wake‐up call for women's bladders!

**DOI:** 10.1002/bco2.70132

**Published:** 2025-12-29

**Authors:** Jingyi Zhou, Donghai Zhang, Ruomeng Bi, Lei Xia, Zengyuan Zhou, Qian Zhou, Yongsheng Yu, Qingmiao Ge, Runtao Zhang

**Affiliations:** ^1^ Department of Clinical and Translational Research Center, Shanghai Key Laboratory of Maternal Fetal Medicine, Shanghai Institute of Maternal‐Fetal Medicine and Gynecologic Oncology, Shanghai First Maternity and Infant Hospital, School of Medicine Tongji University Shanghai China; ^2^ Department of Reproductive Immunology, Shanghai Key Laboratory of Maternal Fetal Medicine, Shanghai Institute of Maternal‐Fetal Medicine and Gynecologic Oncology, Shanghai First Maternity and Infant Hospital, School of Medicine Tongji University Shanghai China; ^3^ Chongqing Institute of Green and Intelligent Technology Chinese Academy of Sciences Chongqing China; ^4^ Chongqing School University of Chinese Academy of Sciences Chongqing China; ^5^ Department of Nephropathy The First Affiliated Hospital of Anhui Medical University Hefei Anhui China; ^6^ Beijing Normal‐Hong Kong Baptist University Zhuhai Guangdong China

**Keywords:** appendicular muscle‐to‐fat ratio, cross‐sectional study, national health and nutrition examination survey, urinary incontinence

## Abstract

**Objective:**

This study aimed to determine the association between appendicular muscle‐to‐fat ratio (aMFR) and the risk of urinary incontinence (UI) in women.

**Methods:**

A total of 4393 participants recruited from the National Health and Nutrition Examination Database (NHANES) from 2011 to 2018 were included in this study. We screened variables using least absolute shrinkage and selection regression, multivariate logistic regression, dose–response curve and nomogram to estimate the relationship between aMFR and UI. The accuracy and discrimination of the nomogram were validated using calibration, receiver operating characteristic (ROC), and decision curve analysis (DCA) curves.

**Results:**

Participants with UI had a lower aMFR than those without (I [0.57, interquartile range [IQR]: 0.49, 0.69] vs 0.63, IQR: 0.54, 0.77, *P* < 0.05). Dose–response curves and multivariate logistic regression showed a negative correlation between the aMFR and the risk of developing UI [adjusted odds ratio (aOR) = 0.35, 95% confidence interval (CI) = 0.226–0.537, *P* < 0.001]. Validation of the calibration curves, ROC curves and DCA curves revealed the good predictive ability of the UI nomogram, and the area under the ROC curve in the predictive model was 0.668 (95% CI = 0.641–0.695) in the training set and 0.660 (95% CI = 0.633–0.687) in the testing set, which demonstrated the good performance of the model.

**Conclusion:**

A low aMFR was significantly associated with an increased risk of UI in women in the US and could be included in risk prediction models for female UI.

## 1 INTRODUCTION

Urinary incontinence (UI) in women refers to involuntary leakage of urine through the urethra.^1^ The current classification of UI includes stress incontinence, urge urinary incontinence, mixed urinary incontinence and urinary incontinence filling^2^. UI has a significant impact on women's physical and mental health as well as their quality of life.^3^ The prevalence of UI varies markedly across countries. The Risk of UI Study conducted by Kaiser et al. revealed that White and Mexican‐American women are approximately 2.5 times more likely to develop stress UI than African American women, after considering factors such as age, parity, body mass index (BMI) and activity levels.^4^, ^5^, ^6^ The incidence of UI in women in China is approximately 30.9%. However, studies exploring the relationship between UI and ethnicity in this context are yet to be conducted. Currently, known risk factors for UI include age, race, marital status, family income level, recreational activities, diabetes and metabolic syndrome.^5^ Additionally, factors such as the number of pregnancies and deliveries, nervous system diseases, obesity and other chronic conditions may also increase the risk of UI through functional or anatomical changes.^7^ Pregnancy and childbirth have long been recognized as the most significant risk factors contributing to female UI. Recently, the focus of research has shifted to the correlation between obesity and UI. Research has confirmed that being overweight or obese plays a significant role in the incidence and 5‐year prevalence of UI. Obesity may increase intra‐abdominal pressure, which in turn may weaken pelvic muscles and innervation.^8^

Previous studies have primarily utilized BMI to evaluate the risk relationship between UI and obesity. Although BMI can reflect overall fatness to some extent, it has limitations in accurately determining the degree of obesity owing to the accumulation of visceral fat.^9^ To address this, we introduced the appendicular muscle‐to‐fat ratio (aMFR) to accurately assess the degree of obesity. aMFR is defined as the ratio of appendicular muscle mass to total body fat mass.^10^ Previous research has demonstrated that aMFR can be useful in assessing the risk of cardiovascular and chronic kidney diseases in older adults.^11^ AnnM et al. initially revealed a link between UI and changes in body composition.^12^ However, the association between UI and aMFR in women remains unclear. Given the complexity of UI diagnosis, understanding its risk factors is crucial. Therefore, our study aimed to develop a risk nomogram that includes aMFR for predicting UI.

## 2 METHODS AND PARTICIPANTS

### 2.1 Design of research

Data were obtained from the national health and nutrition examination survey (NHANES), a nationally representative cross‐sectional survey that collected nationally representative data on population health and nutritional status. The Institutional Review Board of the Ethics Review Board of the National Center for Health Statistics provided ethical clearance for this study using previously collected public data.

**FIGURE 1:**
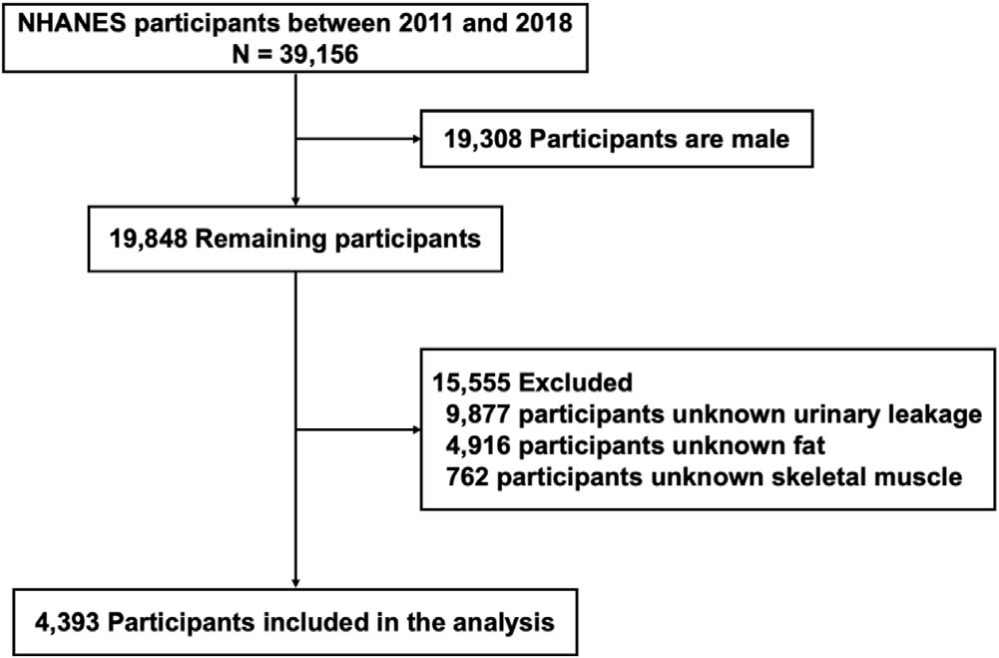
Schematic of the process for the participants in the study and the inclusion and exclusion criteria.

From 2011 to 2018, data from 39 156 participants were used in this survey. The exclusion criteria were as follows: (1) Male participants with UI (*n* = 19 308); (2) participants with unknown UI (*n* = 9877); (3) participants with unknown fat (*n* = 4916); and (4) participants with unknown skeletal muscle mass (*n* = 762). Ultimately, we included 4393 participants for further analysis (Figure 1).

### 2.2 Evaluation of aMFR

Whole‐body dual‐energy X‐ray absorptiometry (DXA) scans were performed on participants by specialized technicians using a Hologic fan‐beam densitometer (Hologic, Bedford, Massachusetts, USA) to measure MFR. The procedures adhered to standards set by the International Society for Clinical Densitometry. Participants were excluded if they were pregnant, had undergone imaging within the past 7 days, or self‐reported a weight exceeding 450 pounds or a height over 6′5″.^13^, ^14^

### 2.3 Study variables and outcome

To enhance the accuracy of our clinical prediction model by examining the relationship between the aMFR and UI in women, we incorporated a comprehensive set of variables that may influence this relationship. These variables included age, race, education, marital status, household income poverty, moderate recreational activity, vigorous recreational activity, hypertension, diabetes, urea nitrogen, serum creatinine level and cotinine level. Detailed instructions for specimen collection and handling are available at the NHANES website (https://www.cdc.gov/nchs/nhanes/). Age, family income poverty, serum creatinine level and cotinine level were considered continuous values. We used the following categorical variables: race (Mexican‐American, other Hispanics, non‐Hispanic White and other race), educational level (≤high school and >high school), marital status (married, unmarried and others), moderate recreational activities (yes or no), vigorous recreational activities (yes or no), hypertension (yes or no) and diabetes (yes or no). Hypertension and diabetes were categorized into ‘yes’ and ‘no’ groups based on the NHANES self‐administered questionnaire.

The outcome of this study was whether the participants had UI. Data were obtained from the NHANES interviews and self‐administered questionnaires. Participants were considered to have UI if they answered ‘yes’ to any of the following questions, including ‘In the past 12 months, have you leaked or lost control of even a small amount of urine with an activity such as coughing, lifting, or exercising?’ ‘In the past 12 months, have you leaked or lost control of even a small amount of urine without an activity such as coughing, lifting, or exercising, or without an urge to urinate?’ ‘In the past 12 months, have you leaked or lost control of even a small amount of urine with an urge or pressure to urinate and can you not get to the toilet quickly enough?’ A consistency check was built into the NHANES CAPI system to minimize data entry errors and ensure the quality and validity of the questions.

### 2.4 Statistical analysis

All data were analysed using R (version 3.5.3) and SPSS (version 24.0) software. A total of 4393 participants were randomly assigned to two groups: 50% were allocated to the training set, and the remaining 50% were assigned to the test set. For continuous variables, we assessed the distribution of data. Normally distributed continuous variables were described using means and standard deviations (SD), whereas nonnormally distributed continuous variables were characterized by medians and interquartile ranges (upper and lower quartiles). Categorical variables are presented as frequencies and percentages. Statistical comparisons were performed using the *t*‐test for continuous variables and the chi‐square test for categorical variables.

LASSO regression is a powerful technique that combines shrinkage and variable selection in linear regression models. The complexity of the LASSO model is regulated by λ. As λ increases, the penalty imposed on the model to include additional variables becomes more stringent. Consequently, this results in a more parsimonious model with fewer predictors. Variables with zero regression coefficients after the contraction process were excluded from the model, whereas variables with nonzero regression coefficients had the strongest correlation with the response variable.^15^ Thus, the influence of uncorrelated independent variables on the model was eliminated as much as possible, and the generalization performance of the model was improved. LASSON performs a validation analysis of the included variables and ultimately selects the best λ value. Therefore, we used the LASSON regression analysis to analyse all participants and included the variables of age, race, education, marital status, family income poverty, moderate recreational activities, vigorous recreational activities, hypertension, diabetes, urea nitrogen, serum creatinine, cotinine and aMFR. Predictive modelling using multivariate logistic regression analysis by introducing the features selected in the LASSO regression model, we characterized the features using odds ratios (ORs) with 95% confidence intervals (CI) and *P*‐values.

Several validation methods were used to further assess the accuracy and goodness‐of‐fit of the nomogram. The area under the receiver operating characteristic (ROC) curve can help determine the optimal diagnostic threshold that makes the best classification. Clinicians can use the Optimal Operating Point (OOP) or Youden index on the ROC curve to select a threshold that maximizes both sensitivity and specificity, thus improving diagnostic accuracy. Decision curve analysis (DCA) curves were used to characterize the net benefits based on different risk thresholds. Calibration curves were used to evaluate calibration of the UI risk nomogram.

Differences were considered statistically significant at *P* < 0.05. The statistical significance level was two sided. All introduced variables were statistically analysed for significance levels, and statistically significant variables were applied to construct nomogram prediction models.

## 3 RESULTS

### 3.1 Characteristics of participants

A total of 4293 subjects participated in the current statistical analysis, using data from the NHANES 2011–2018. Our study included 1039 (24.2%) UI participants and 3254 (75.8%) non‐UI participants (Table 1). The mean age of all participants was 40 years [interquartile range [IQR]: 29–49], and 37.5% were non‐Hispanic White. The UI and non‐UI participants differed significantly in terms of age, race, education, marital status, family income poverty, moderate recreational activities, vigorous recreational activities, hypertension, diabetes, blood urea nitrogen, serum creatinine, cotinine and aMFR (all *P* < 0.05). Participants with UI were more likely to be older, non‐Hispanic White, married, engaged in moderate and vigorous recreational activities and have diabetes. Compared with participants without UI, participants with UI tended to have higher blood urea nitrogen, serum creatinine and cotinine levels. It is worth mentioning that participants with UI may have a lower aMFR than the control group (0.57, IQR: 0.49, 0.69, *P* < 0.0017 vs. 0.63, IQR: 0.54, 0.7).

**TABLE 1:** Baseline characteristics of NHANES participants between 2011 and 2018 (*n* = 4293).^a^

Characteristic	All	Non‐ urinary incontinence	Urinary incontinence	*P*‐value
	Patients	No. (%)	No. (%)	
	*N* = 4293	*N* = 3254 (75.8)	*N* = 1039 (24.2)	
Age				<0.001
Mean (IQR)	40.00 (29.0, 49.0)	38.00 (28.0, 47.0)	46.00 (34.0, 53.0)	<0.001
Race				<0.001
Mexican‐American	639 (14.9)	475 (14.6)	164 (15.8)	
Other Hispanic	457 (10.6)	357 (11.0)	100 (9.6)	
Non‐Hispanic White	1608 (37.5)	1217 (37.4)	391 (37.6)	
Non‐Hispanic Black	853 (19.9)	604 (18.6)	249 (24.0)	
Other race	736 (17.1)	601 (18.5)	135 (13.0)	
Education				<0.001
≤High school	1479 (34.5)	1065 (32.7)	414 (39.8)	
High school	2814 (65.5)	2189 (67.3)	625 (60.2)	
Marital status				0.030
Married	2018 (47.0)	1560 (47.9)	458 (44.1)	
Unmarried and others	2275 (53.0)	1694 (52.1)	581 (55.9)	
Family income poverty				<0.001
Mean (SD)	2.13 (1.06, 4.09)	2.22 (1.09, 4.19)	1.86 (0.94, 3.80)	<0.001
≤1.5	1629 (37.9)	1182 (36.3)	447 (43.0)	
1.5–3.5	1305 (30.4)	1005 (30.9)	300 (28.9)	
3.5	1359 (31.7)	1067 (32.8)	292 (28.1)	
Moderate recreational activities				<0.001
Yes	3142 (73.2)	2316 (71.2)	826 (79.5)	
No	1151 (26.8)	938 (28.8)	213 (20.5)	
Vigorous recreational activities				0.002
Yes	2298 (53.5)	1699 (52.2)	599 (57.7)	
No	1995 (46.5)	1555 (47.8)	440 (42.3)	
Hypertension				<0.001
Yes	3322 (77.4)	2604 (80.0)	718 (69.1)	
No	971 (22.6)	650 (20.0)	321 (30.9)	
Diabetes				<0.001
Yes	3963 (92.3)	3052 (93.8)	911 (87.7)	
No	330 (7.7)	202 (6.2)	128 (12.3)	
Urea nitrogen (mg/dL)	11.00 (9.0, 14.0)	11.00 (9.0, 14.0)	12.00 (9.0, 14.0)	<0.001
Serum creatinine (mg/dL)	0.71 (0.6, 0.8)	0.71 (0.6, 0.8)	0.72 (0.6, 0.8)	0.009
Cotinine (ng/mL)	0.03 (0.01, 1.35)	0.03 (0.01, 0.63)	0.04 (0.01, 23.60)	0.007
aMFR	0.62 (0.52, 0.75)	0.63 (0.54, 0.77)	0.57 (0.49, 0.69)	<0.001

Abbreviations: aMFR, appendicular muscle mass‐to‐total body fat ratio; IQR, interquartile range; SD, standard deviation.

^a^ For categorical variables, *P*‐values were analysed using the chi‐square test. For continuous variables, the *t*‐test for slope was used in the generalized linear models.

### 3.2 Association between the aMFR and urinary incontinence

**FIGURE 2:**
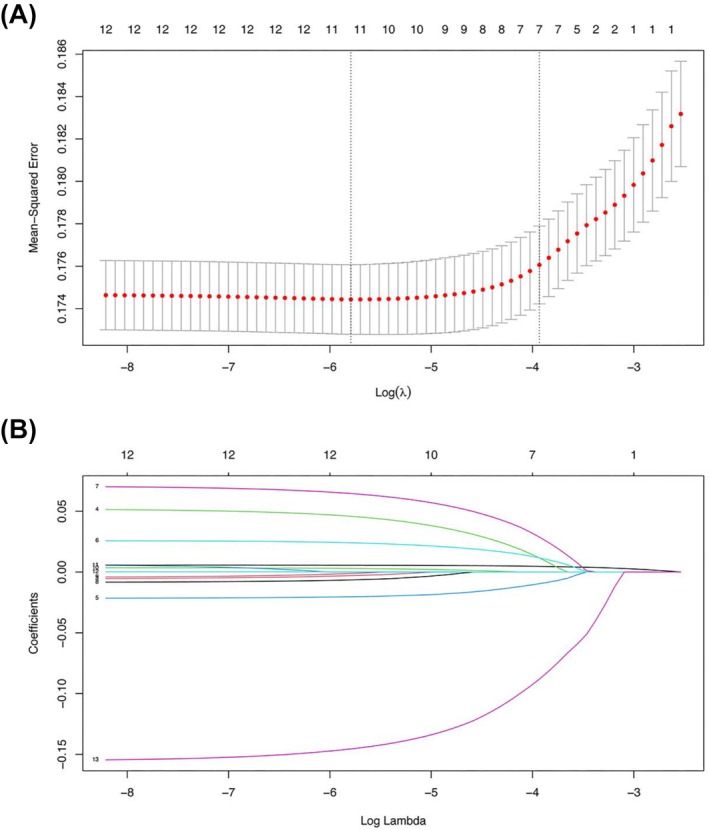
Screening by including variables in Least Absolute Shrinkage and Selection Operator (LASSO) regression. Plot the partial likelihood deviation (binomial deviation) curve with respect to log(λ) by validating the optimal parameter (λ) in the LASSO model and plot the dashed vertical line according to the 1 standard error criterion (A). Variable selection based on the LASSO binary logistic regression model. A coefficient profile is generated from the log (lambda) series (B). Ten variables with nonzero coefficients are selected from the optimal lambda.

**FIGURE 3:**
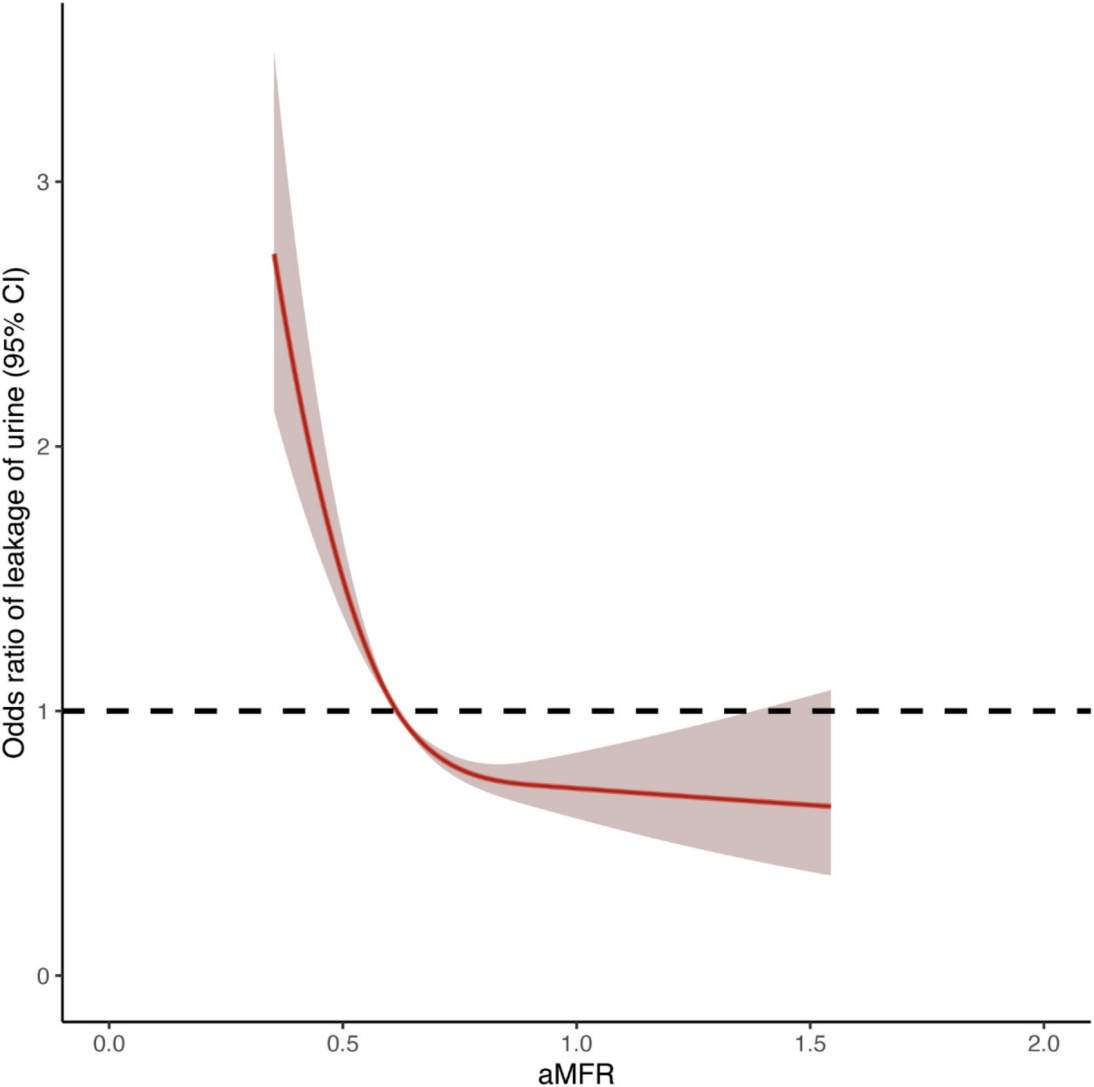
Dose–response analysis between appendicular muscle‐to‐fat ratio (aMFR) and urinary incontinence (UI) risk in women. Dose–response curves show a negative correlation between aMFR and urinary incontinence (UI).

**FIGURE 4:**
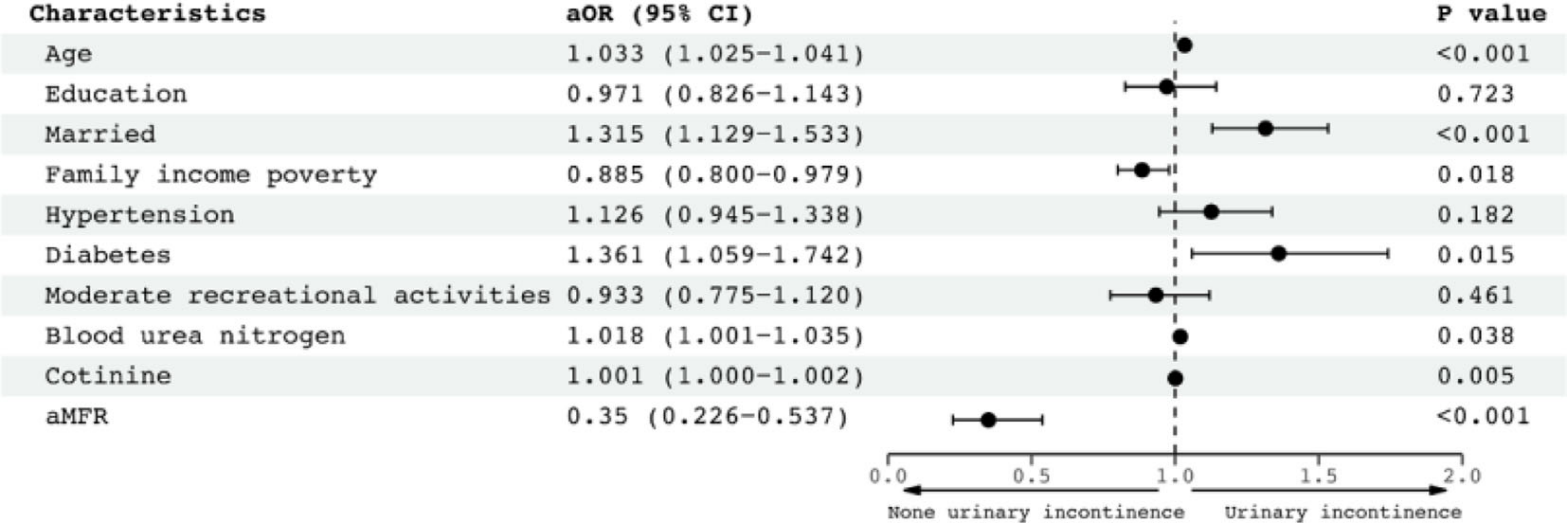
Multivariate logistic regression analysis of the association of age, education, married, family income poverty, hypertension, diabetes, moderate recreational activities, blood urea nitrogen, cotinine and appendicular muscle‐to‐fat ratio (aMFR) with urinary incontinence (UI).

Of the 13 variables with relevant characteristics, 10 nonzero coefficient variables were selected by LASSO regression, which were significantly correlated with UI (Figure 2). These variables included age, marital status, family income poverty, diabetes, urea nitrogen level, cotinine level and aMFR. The dose–response curves showed a negative correlation between aMFR and UI (Figure 3). Finally, multivariate logistic regression analyses of the 10 variables identified seven autonomous risk factors associated with UI. These seven factors were included in the nomogram. As shown in Figure 4, multivariate logistic regression analysis indicated that participants with low aMFR levels were significantly associated with an increased risk of UI [adjusted odds ratio (aOR) = 0.35, 95% confidence interval (CI) = 0.226–0.537, *P* < 0.001].

### 3.3 Prediction model development and validation

**FIGURE 5:**
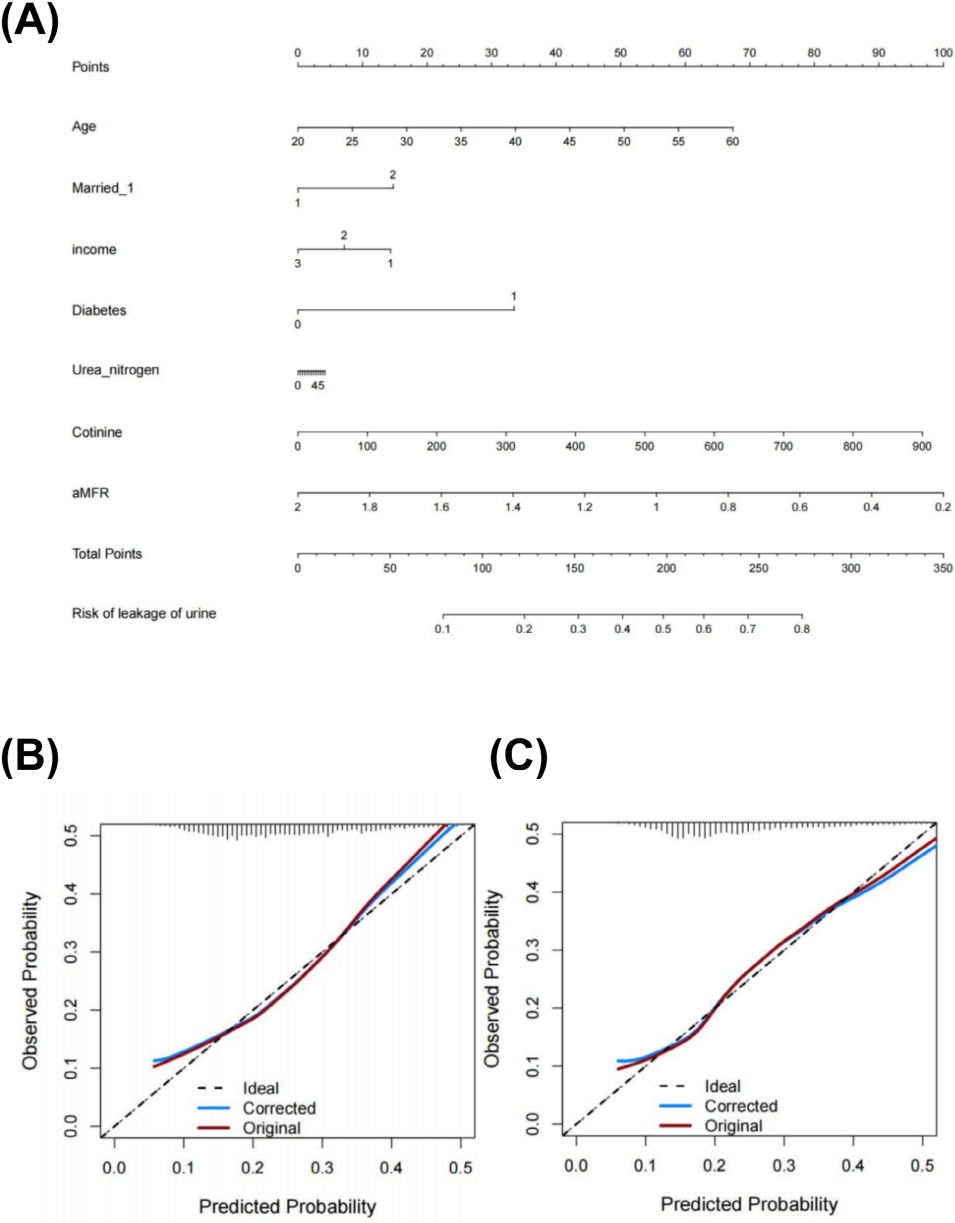
Nomogram for predicting the risk of urinary incontinence (UI) (A). Calibration curve for the nomogram of training set (B). Calibration curve for the nomogram of testing set (C).

**FIGURE 6:**
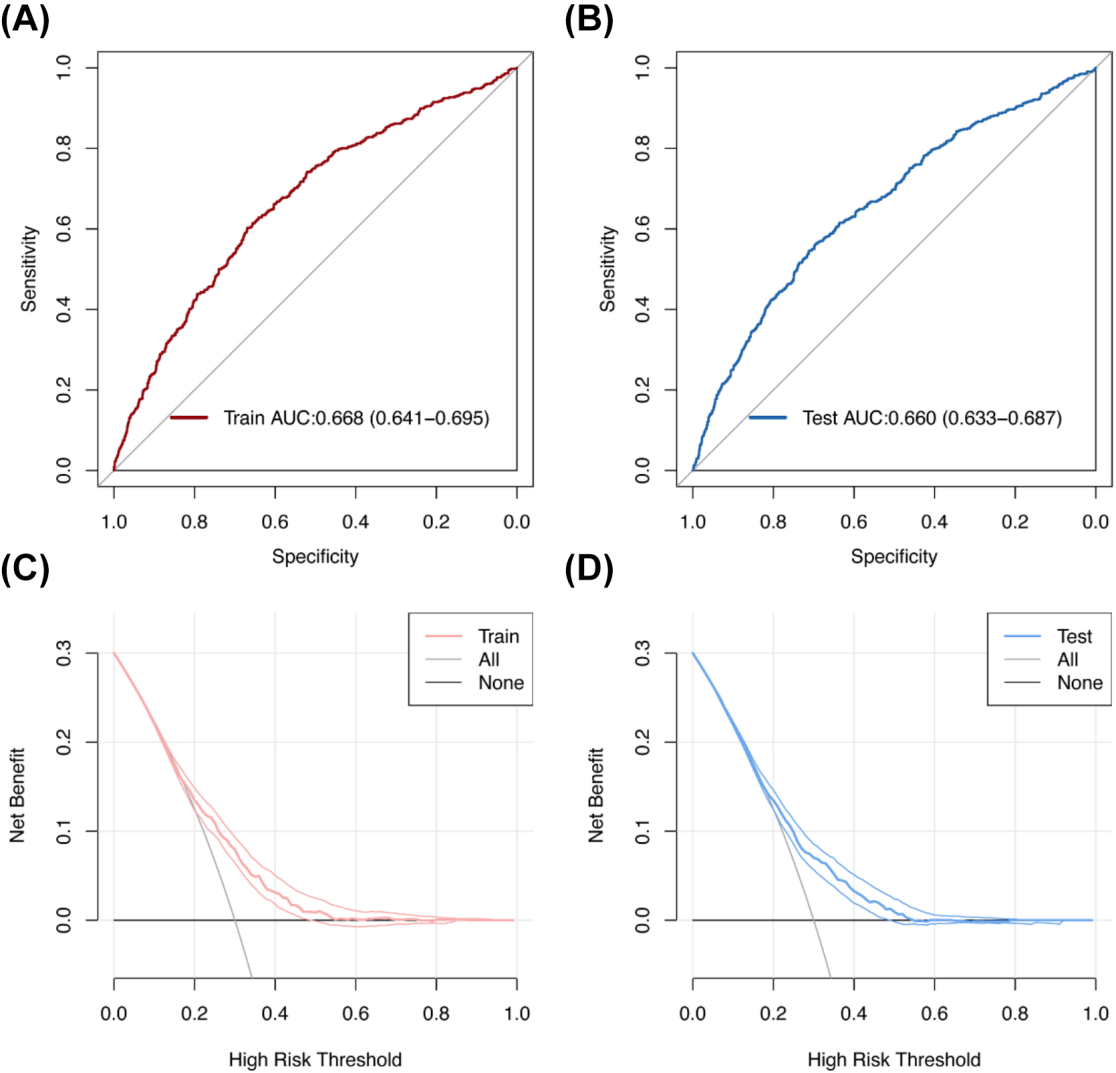
Receiver operating characteristic (ROC) validation of the UI risk nomogram prediction in training set (A) and in testing set (B). Decision curve analysis (DCA) of nomogram in training set (C) and testing set (D).

After introducing these 10 independent characteristics, we constructed a UI predictive risk model (Figure 5A). To better predict the accuracy of the model, 50% of the participants were randomly selected as the training set, and the remaining participants were selected as the testing set. The calibration curves showed that the actual and predicted incidence rates of the model were in general agreement. The model had good prediction ability in both the training and test sets (Figure 5B, C). The area under the ROC curve in the predictive model was 0.668 (95% CI = 0.641–0.695) in the training set and 0.660 (95% CI = 0.633–0.687) in the testing set, which demonstrated the good performance of the model (Figure 6A, B). Summarizing the validation methods above, the model has good prediction ability. Furthermore, the decision curve proved that the model had a desirable net benefit in both the training and testing sets (Figure 6C, D).

## 4 DISCUSSION

Nomograms are highly regarded as reliable and practical predictive tools. They are capable of quantifying individual risks by integrating various prognostic and decision‐making variables. This integration allows the generation of individual probabilities of clinical events, thereby incorporating multiple important prognostic factors into a cohesive model.^16^ In previous studies, clinical prediction models and simple scoring charts for the risk of urinary incontinence following percutaneous nephrolithotripsy have been reported.^17^ Prior research has also utilized ultrasound outcome measures and clinical characteristics to assess urinary incontinence in pregnant women.^18^ While most existing prediction models for urinary incontinence focus on estimating the probability of its occurrence following clinical interventions, few have comprehensively organized, analysed and constructed a relatively complete clinical prediction model for the development of urinary incontinence.

We conducted a cross‐sectional study using the NHANES database from 2011 to 2018 and found that aMFR was an independent risk factor for UI and negatively associated with its occurrence. Based on these findings, we constructed a clinical prediction model for urinary incontinence that incorporated several key variables: age, marital status, household income poverty, diabetes, blood urea nitrogen level, blood creatinine level and aMFR values. The predictive performance of the model was evaluated using calibration curves, ROC curves and DCA curves, and it demonstrated good predictive properties. Urinary incontinence significantly affects the quality of life of female patients and can cause considerable psychological distress. Therefore, early identification of urinary incontinence can facilitate preventive treatment for those at a higher risk, potentially improving their overall well‐being and reducing the burden associated with this condition.

The causes of urinary incontinence are multifactorial, with muscle weakness being the primary factor contributing to its onset in women. This is largely associated with relaxation of the pelvic floor muscles and urethral sphincter, which may be influenced by age and obesity.^19^ A longitudinal study based on a Chinese population revealed that high BMI can negatively impact the prognosis of urinary incontinence.^20^ Numerous studies have consistently demonstrated that obesity is a significant, independent risk factor for both the prevalence and incidence of urinary incontinence.^21^, ^22^ Evidence suggests a strong dose–response relationship between BMI and urinary incontinence, with a 5‐unit increase in BMI correlating to a 20% to 70% increase in risk.^23^ Moreover, the maximum effect of body weight in well‐controlled analyses rarely exceeded the odds ratio (OR) of 4 to 5. A meta‐analysis on the factors contributing to the development of urinary incontinence in older women worldwide suggested that obesity may accelerate UI progression of urinary incontinence.^24^, ^25^ The mechanism underlying obesity‐induced urinary incontinence has been validated in animal models. Wang et al. found that obese rats (Zucker fatty rats) exhibit insulin resistance, increased frequency of urination and decreased leak point pressure (LPP) compared to controls (Zucker lean rats),^26^ in addition to increased intramuscular lipid deposition in urethral transverse muscle fibres in obese rats, suggesting that obesity may contribute to urinary incontinence by affecting urethral sphincter function,^27^ thus providing insight into whether obesity and muscle loss or weakness may increase the risk of developing urinary incontinence.

Visceral fat accumulation is associated with decreased muscle function as it may result in altered muscle morphology and decreased neural adaptations. Salagre et al. verified using an obese mouse model that fat accumulation may cause altered mitochondrial fission, fusion and autophagy in muscle cells, leading to mitochondrial dysfunction and decreased muscle function.^28^ Xiang et al. preliminarily verified that obesity‐induced myasthenia gravis might be related to the SIRT1 pathway.^29^ In the past, BMI was mostly used to determine obesity and overweight; however, BMI alone cannot be used to assess obesity because of its individual heterogeneity.^30^ The location of fat accumulation varies among individuals, with some primarily accumulating subcutaneous fat, and others predominantly accumulating visceral fat. This variation can lead to different prognoses in several metabolism‐related diseases.^31^, ^32^

The aMFR can assess the ratio of appendicular muscle content to total body fat content. With the combination of DXA or bio‐electrical‐impedance analysis (BIA), we can better assess aMFR values to obtain a more accurate rate of visceral fat accumulation.^31^, ^33^ The current study found that alterations in aMFR are associated with cardiovascular disease, type 2 diabetes mellitus and osteoporosis in perimenopausal women.^34^, ^35^, ^36^ However, an association between aMFR and urinary incontinence in women has not been previously reported. To the best of our knowledge, this is the first study to reveal this association.

However, this study had several limitations. First, as a cross‐sectional study based on the US NHANES database, we were unable to establish a causal relationship between urinary incontinence and reduced aMFR. Further validation and research are required to explore this potential causal link. Second, our analysis only considered some common clinical characteristics as predictors. Other unconsidered factors may influence the risk of urinary incontinence. As our understanding of UI pathogenesis of urinary incontinence evolves, we can incorporate additional predictor variables to enhance the completeness and accuracy of clinical prediction models. Third, all data were derived from a US database, which may limit the generalizability of our findings to other populations. At present, the following theories are widely accepted in clinical practice with regard to urinary incontinence in the elderly: Firstly, the decline in muscle function that occurs with age can result in pelvic floor muscle laxity. Secondly, the presence of neurological dysfunction can result in impairment to the nerves that regulate the pelvic muscles. Another type of incontinence, termed mixed urinary incontinence, results from a combination of the aforementioned causes.^37^

Geographic specificity of the data should be considered when interpreting the results. Fourth, although we employed LASSO regression and multivariate logistic regression methods to control for confounding bias as much as possible, there may still be potential confounders that were not included in our analysis. These unmeasured confounders may have inevitably affected the study results. Additionally, it was challenging to clearly interpret the association between aMFR and the three types of urinary incontinence, as the NHANES database does not explicitly classify these types of incontinence. Urinary incontinence in the NHANES is primarily diagnosed through questionnaires, which may introduce some limitations in the interpretation of our findings. Given these limitations, we acknowledge that the nomogram presented in this study has limitations in its applicability and accuracy.

## 5 CONCLUSION

In conclusion, there was an association between low aMFR and the occurrence of UI. A low aMFR may serve as a predictor of UI. This finding underscores the importance of considering the aMFR in the assessment and management of UI, particularly in high‐risk populations.

## AUTHOR CONTRIBUTIONS

Jingyi Zhou, Qingmiao Ge and Donghai Zhang screened the datasets in the database. Jingyi Zhou and Qingmiao Ge conducted preliminary data analysis. Ruomeng Bi, Runtao Zhang and Zengyuan Zhou validated the data accuracy. Jingyi Zhou and Ruomeng Bi wrote the first draft of the manuscript. Jingyi Zhou, Runtao Zhang, Donghai Zhang and Zengyuan Zhou wrote the final version of the manuscript. Yongsheng Yu and Qian Zhou polished the manuscript and provided guidance on its completion. All authors have read and approved the final manuscript.

## CONFLICT OF INTEREST STATEMENT

The authors declare that they have no conflict of interests in this project.

## CONSENT FOR PUBLICATION

Not applicable.

## Data Availability

The datasets generated for this study are available on request to the corresponding author.
